# Case Report: acute and long-term effects of a massive beta-mercaptoethanol inhalation

**DOI:** 10.3389/ftox.2026.1916362

**Published:** 2026-08-06

**Authors:** Marion Bernard, Philippe Van Brussel, Cécile Lardinois, Virginie Doyen, François M. Carlier, Thomas Planté-Bordeneuve

**Affiliations:** 1 Department of Pneumology, CHU UCL Namur, Yvoir, Belgium; 2 Service de Prévention et Protection Au Travail-CESI, Brussels, Belgium; 3 Department of Neurology, CHU UCL Namur, Yvoir, Belgium; 4 Pole of Lung, Nose and Skin (LUNS), Institut de Recherche Expérimentale et Clinique (IREC), Université Catholique de Louvain, Brussels, Belgium

**Keywords:** beta-mercaptoethanol, case report, irritant-induced asthma, reactive airway dysfunction syndrome, toxicity

## Abstract

**Introduction:**

β-mercaptoethanol is widely used in biochemistry and industrial applications. Animal toxicity has been demonstrated but very limited data exists regarding its human effects.

**Case Description:**

A 40-year-old male patient was exposed to β-mercaptoethanol vapours after an accidental occupational spillage. He developed dizziness, blurred vision, a headache and epistaxis within minutes alongside dyspnoea and cough in the following hours. Lung function tests displayed bronchial hyperreactivity and increased resistances, consistent with a reactive airways dysfunction syndrome. Neuropsychological testing showed memory difficulties affecting both working and long-term verbal memory, together with attentional and executive weaknesses. Twenty months after the incident, the patient still experienced memory impairment and dyspnoea while bronchial hyperreactivity remained present.

**Conclusion:**

Respiratory exposure to large quantities of β-mercaptoethanol vapours can cause reactive airways dysfunction syndrome and is associated with long-term neurological and respiratory symptoms.

## Introduction

β-Mercaptoethanol (BME, (HOCH2CH2SH; CAS No. 60–24-2)) is a sulfur-containing reducing agent which cleaves disulfide bonds in proteins resulting in their denaturation. In addition, it possesses anti-oxidant properties and can act as a chain transfer agent in polymerization. It is widely used in molecular biology and biochemistry well as in the dyestuff, plastic and rubber industry. It is a transparent, volatile thiol with a strong odour (Ruth, 1986) and contact with skin or mucosal surfaces can cause irritation while its ingestion can be lethal (Eriksson et al., 1989). Despite its widespread use, no data is available regarding its potential human respiratory toxicity. Here, we report a case of an accidental workplace inhalation exposure to BME vapors resulting in the development of a reactive airways dysfunction syndrome (RADS).

## Case description

**FIGURE 1:**
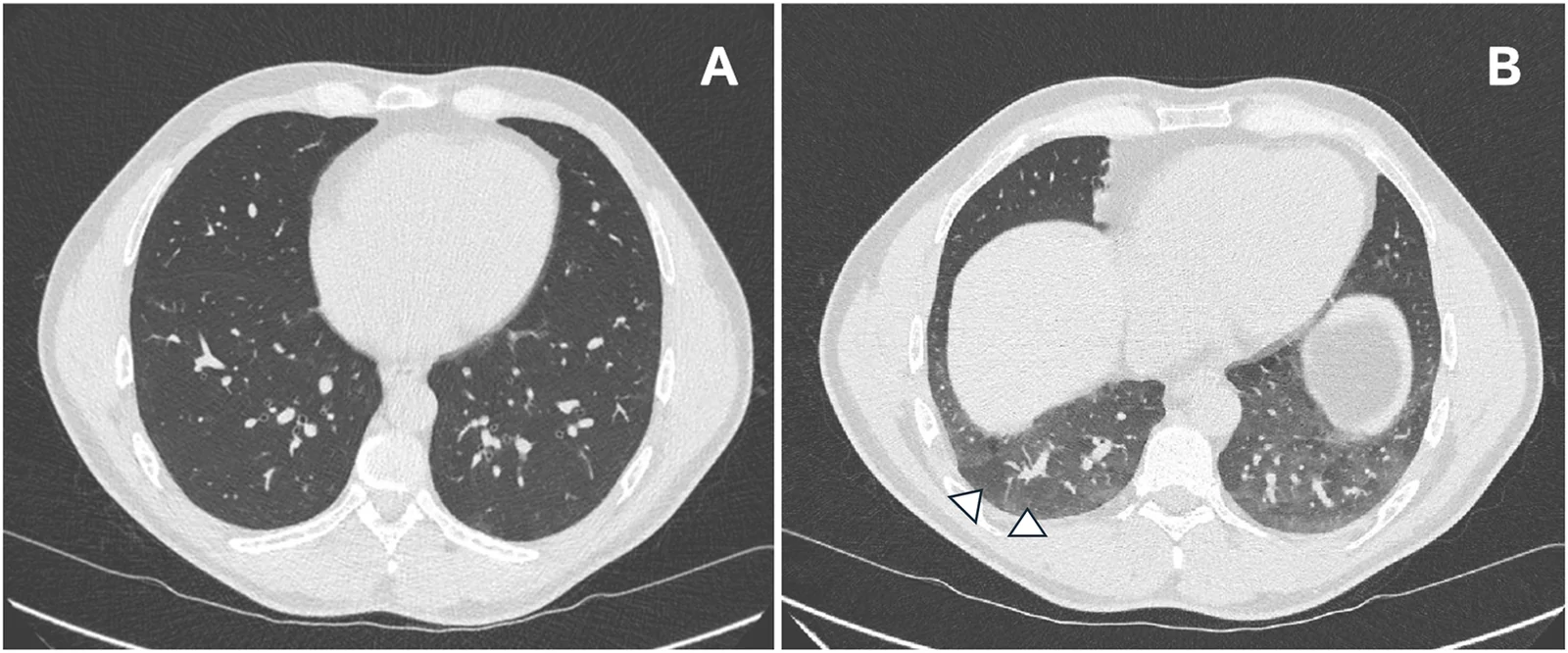
Axial images of the thorax computed tomography 1 month after the exposition, on inspiratory phase **(A)** and expiratory phase **(B)**. A mosaic pattern, consistent with small airway disease, of the lower lobes is present on the expiratory acquisition (differential attenuation, arrowheads).

A 40-year-old non-smoking male patient with no relevant medical history was working as a process operator on a production line in a biopharmaceutical company when 4 L of 100% concentrated BME were accidentally spilled into a stainless-steel container filled with dry ice, leading to the uncontrolled release of BME vapours. The patient noticed the characteristic smell of BME for a few seconds before complete abrogation of this stimulus. Approximately 30 minutes after the incident, he was finally removed from the contaminated environment by colleagues who had noticed the strong odour. Later estimation of the maximum exposition levels using the Stoffenmanager tool (Stoffenmanager®, version 8.3), allowing quantitative estimation of workplace exposure, evaluated a maximum atmospheric concentration of 159 mg/m^3^ (of note, no safe acute exposition concentrations have been defined, but the workplace environment exposure level has been proposed to be no more than 0.64 mg/m^3^). Within minutes after his removal, he developed dizziness, blurred vision, xanthopsia, a headache and epistaxis. He was brought to the closest emergency department, where his vital parameters were within normal limits (blood pressure 130/70 mmHg, pulse 75 beats per minute, oxygen saturation at room air 97%, temperature 36.0 °C). His physical examination at that time was characterized by the presence of an erythematous pharynx while his cardiopulmonary auscultation was unremarkable. His bloodwork was within normal range for inflammatory, renal, hepatic and muscular parameters and a chest X-ray did not show any significant abnormalities. Within a few hours, the patient experienced a modified Medical Research Council (mMRC) grade 1 dyspnoea, as well as slightly productive cough. In the first 24 h, the patient developed short-term memory impairment and insomnia, impacting his daily activities. He was evaluated at our outpatient clinic 12 days after the incident, mentioning the presence of a persistent mMRC grade 1 dyspnoea, substernal chest pain on inspiration, scant clear sputum and new-onset hoarseness. His physical examination was within normal limits, and his saturation was 99% at room air. His neurological examination was likewise unremarkable. Routine laboratory studies, including liver function tests, were within normal limits. Pulmonary function tests were within reference values (Table 1). His fractional exhaled nitric oxide was measured at 29 parts per billion ((ppb) (elevated >50ppb, intermediate 25–50 ppb (Dweik et al., 2011)). Forced oscillation technique (FOT) revealed moderately elevated airway resistances (R_tot_5 Hz 167% of the predicted value). A histamine challenge test was performed and the provocation concentration inducing a 20% fall in forced expiratory volume in one second (FEV_1_ PC_20_) was 1.0 mg/mL (normal >16 mg/mL (Coates et al., 2017)). High resolution computed tomography of the chest demonstrated a mosaic attenuation pattern with air trapping at the lung bases on expiratory acquisitions (Figure 1).

**TABLE 1:** Results of the lung function tests during follow-up.

Time (months)	FEV1, L (% predicted)	FVC, L (% predicted)	FEV1/FVC, % (% predicted)	FeNO (ppb)	PC20 (mg/mL)	R_tot_5 Hz, cmH2O.s/L (% predicted)
0.3	4.36 (104)	5.90 (112)	73 (92)	29	NA	NA
1	4.2 (100)	5.94 (113)	71 (86)	22	0.99	4.76 (167)
4	4.11 (96)	5.54 (106)	74 (92)	NA	0.54	4.06 (141)
7	4.39 (104)	5.84 (110)	75 (93)	NA	0.53	7.02 (249)
8	4.36 (103)	5.83 (110)	75 (93)	20	NA	NA
20	4.61 (110)	6.21 (118)	74 (93)	58	NA	3.70 (137)

The forced expiratory volume at 1 s (FEV1), forced vital capacity (FVC) and the FEV1/FVC, index remained within normal range. Bronchial hyperreactivity was evaluated through the histamine provocation test, with the concentration resulting in a 20% fall of the FEV1 (PC20) remaining low over time (normal >16 mg/mL). The Fractional exhaled nitric oxide (FeNO), typically increased type-2, inflammation, was elevated at the last visit (high>50ppb). The respiratory resistance measured at a frequency of 5 Hz by forced oscillometry (R_tot_5 Hz) remained elevated over time. Data are presented as absolute measures (L) and as percentages of the predicted value. NA, not available.

The electroencephalogram was normal, whereas neuropsychological testing revealed memory difficulties affecting both working memory and long-term verbal memory, together with attentional and executive weaknesses. Brain MRI showed a few faint, nonspecific T2 FLAIR hyperintensities of the subcortical white matter without other lesions. Of note, the patient also reported a visual decline; ophthalmologic evaluation confirmed reduced visual acuity with a normal eye fundus and corrective lenses were prescribed.

Taken together, the abrupt onset after a single high-level respiratory exposure, the absence of prior respiratory disease, and the presence of nonspecific bronchial hyperresponsiveness to histamine with the persistence of symptoms were consistent with a Reactive Airways Dysfunction Syndrome (RADS) secondary to acute BME inhalation. Treatment was initiated at the first consultation with a combination of inhaled corticosteroids and a long-acting β2-agonist (beclomethasone 100 mcg/formoterol 6 mcg). The patient also underwent cognitive rehabilitation to aid in his social and professional reintegration, which resulted in some symptomatic improvement, although attentional fatigability and working memory complaints persisted.

Patient’s follow-up took place at 1-, 4-, 8-, and 20-month after the accident. His exertional dyspnoea remained present at an mMRC1 grade with little improvement from the inhaled therapy, and daily activities were still impacted by persistent difficulties in memorization. His pulmonary function tests remained stable and within the normal range, with no evidence of an obstructive ventilatory defect (Table 1). Nonetheless, he consistently displayed elevated resistances on FOT and stable persistent non-specific bronchial hyperreactivity with PC_20_ values of 0.5 mg/mL at 7 months.

## Discussion

BME is a sulfur-containing alcohol solvent of the mercaptan family, used in biochemistry for its antioxidant effect and protein denaturing properties. It is a volatile thiol with a strong odour, with a low threshold of smell detection of 0.4–2 mg/m^3^, alongside marked mucosal irritant effects (Ruth, 1986; White et al., 1973). This theoretically protects subjects against massive inhalation as spillage is rapidly noticed. Despite its widespread use, limited data exists regarding its human toxicity. Indeed, a single case of human poisoning with BME has been reported following intentional ingestion of approximately 100 mL, which proved fatal (Eriksson et al., 1989). The proposed mechanism of toxicity was inhibition of cytochrome oxidase, the terminal enzyme of the electron transport chain, resulting in histotoxic anoxia (Eriksson et al., 1989). Animal studies have established cutaneous, central nervous system, respiratory, cardiovascular, and hepatic toxicities (White et al., 1973; Organisation for Economic Co-operation and Development, 2005). The only human occupational exposure guideline is proposed by the American Industrial Hygiene Association (AIHA) at 0.2 ppm (0.64 mg/m^3^) for chronic exposure; no acute or respiratory exposure standard exists. By extrapolation, limits are often tripled (Hashimoto et al., 2018), corresponding here to 1.92 mg/m^3^. Our patient’s estimated high-end exposure was approximately 83 times higher than this pragmatic threshold, thus likely corresponding to a harmful concentration.

RADS, or acute irritant-induced asthma (IIA), is defined by the abrupt onset of asthma-like symptoms following a single high-level exposure to an irritant substance and usually appears in subjects with no prior history of respiratory disease (Vandenplas et al., 2014). Importantly, RADS represents only one phenotype of IIA, as multiple moderate to high-level exposures may lead to the development of asthma with a sometimes more insidious onset (Vandenplas et al., 2014). Acute-onset IIA accounts for approximately 5%–14% of occupational asthma cases (Lemiere et al., 2022; Kopferschmitt-Kubler et al., 2021). A wide range of substances has been identified as causal agents for acute irritant induced asthma, with the most commonly implicated being chlorine and its derivatives, nitrogen oxide, ammonia as well as paint fumes (Lemiere et al., 2022; Walters and Huntley, 2020). The case presented here is, to our knowledge, the first to be described following the inhalation of BME following an accidental occupational exposure and as such contributes to the clinical knowledge regarding its potential respiratory toxicity.

In contrast to sensitizer-induced occupational asthma, there is no immunological sensitisation or latency period in RADS. The pathophysiology is not fully elucidated due to limited histopathologic data, but encompasses epithelial denudation from the irritant injury, activation of non-adrenergic and non-cholinergic pathways and airway inflammation with bronchial wall oedema (Lemiere et al., 2022; Dykewicz, 2009). A fibrinohemorrhagic exudate with lymphocytic infiltration can subsequently be seen, followed by signs of epithelial regeneration, ultimately ending in (partial) resolution of bronchial injuries (Lemière et al., 1997; Takeda et al., 2009). The most commonly reported symptoms include cough, dyspnoea and chest tightness (Lemiere et al., 2022) but upper airway involvement and conjunctivitis are also frequently present (Lantto et al., 2022). These typically develop within minutes to hours of exposure and may persist for months or longer. RADS can be associated with persistent symptoms, as up to 39% of patients presented with uncontrolled asthma after a median follow-up of 6.8 years in a recent study (Lantto et al., 2024). Diagnosis relies on the presence of the criteria initially proposed by Brooks et al. (1985), which were revised by the European Academy of Allergy and Clinical Immunology (EAACI) in 2014 (Vandenplas et al., 2014). A close temporal relation with an acute exposure event should be present, and the presence of reversible airflow limitation or non-specific bronchial hyperresponsiveness to histamine or metacholine should be documented (Vandenplas et al., 2014). Importantly, normal spirometric values are present in a majority of patients at baseline (Lantto et al., 2024), emphasizing the importance of challenge tests. Chest X-ray can be useful to exclude other conditions linked with acute exposure such as pulmonary edema but does not contribute to the diagnosis of RADS. Similarly, HRCT is used to assess alternative pulmonary causes to the symptoms and its characteristics in RADS are poorly defined. Findings may encompass trapping and airway thickening, as described in a cohort of symptomatic firefighters who had been exposed to intense dust exposure at the World Trade Center site (Liu et al., 2019). There is currently no standardized protocol for managing RADS. Treatment mirrors asthma therapy, relying on inhaled corticosteroids and bronchodilators, although the response to β2-agonists may be attenuated and most patients require high inhaled corticosteroids doses (Lantto et al., 2022; Gautrin et al., 1994). Even though the timing of the inhaled therapy does not seem to influence disease course, as patients who are started on inhaled corticosteroids more than 1 week after the causal event do not show worse characteristics after 6–8 months (Lantto et al., 2022), prompt initiation should be sought after. Treatment with oral corticosteroids can be given in selected cases, although their use is based on poor evidence (Vandenplas et al., 2014). Limited data exists regarding the prognosis of RADS, with reports of improved bronchial hyperresponsiveness even after months (Malo et al., 1994) alongside persistent symptoms after several years (Lantto et al., 2024). As the underlying mechanisms are not based on sensitization, patient can theoretically return to their work environment. Appropriate measures to avoid (high-dose) exposures to irritants should be put in place and adequate patient education and follow-up provided (Lemiere et al., 2022). Nonetheless, the consequence of IIA are often important as it is associated with reduced work ability and loss of income (Vasankari et al., 2024).

Strikingly, the FeNO, a marker of eosinophilic inflammation, of our patient was not elevated at the initial evaluation and was increased at the 20-month visit. Nonetheless, long term follow-up of an acute and subacute irritant induced asthma cohort showed that 61% of patients had at least one elevated marker of eosinophilic inflammation several years after the index event (Lantto et al., 2024). This hints at a potential participation of type-2 inflammation during the prolonged course of the disease. Furthermore, persistent contact with irritants could also result in progressive sensitization and eosinophilic inflammation after returning to his work environment.

BME has been shown to induce neurological toxicity in mice, resulting in clonic convulsions at high doses and hypoactivity and coma at lower doses (White et al., 1973). There are currently no reports of human neurotoxicity for BME, but central nervous system toxicity has been described with other mercaptans, especially methanethiol (Shults et al., 1970). Toxicity could be related to inhibition of cytochrome oxidase, resulting in cellular anoxia, as has been described with mercaptans (Eriksson et al., 1989).

Several limitations are to be acknowledged. Firstly, as this article reports a single case, its generalizability is limited and most likely only describes part of the possible spectrum related to BME inhalational exposure. Secondly, measurements of BME or its metabolites in blood or urine is not routinely performed or easily accessible. As a consequence, it remains uncertain to what extent the latter were present. Ingestion of BME has been shown to result in the production of 2-mercaptoacetate and inorganic sulfates (Eriksson et al., 1989), which could have contributed to the observed toxicity. In addition, animal studies suggest a role for toxic metabolites as pre-administration of ethanol, a potential competitor for oxidation, before intraperitoneal BME injection in mice was associated with reduced lethality (White et al., 1973). Nonetheless, the direct contact between BME and the respiratory mucosa alongside the chronology supports a direct mechanism of toxicity of the molecule at least at this level.

In conclusion, we report the first case of acute human inhalation toxicity of BME, highlighting its potential long-term respiratory and neurological effects and extending the list of agents associated with RADS.

## Data Availability

The original contributions presented in the study are included in the article/supplementary material, further inquiries can be directed to the corresponding author.
